# Editorial for the Special Issue “Advances and Applications of Polymer Gels for Subsurface Energy and Storage”

**DOI:** 10.3390/gels12090821

**Published:** 2026-09-07

**Authors:** Baojun Bai, Jingyang Pu

**Affiliations:** 1 Department of Earth Sciences and Engineering, Missouri University of Science and Technology, Rolla, MO 65409, USA; 2 College of Energy Innovation, China University of Petroleum (Beijing), Beijing 102249, China

**Keywords:** polymer gels, subsurface energy, improved oil recovery, drilling fluid, CO_2_ geological storage

## Abstract

Polymer gels are essential functional materials for subsurface energy operations, playing a critical role in conformance control, fluid diversion, hydraulic fracturing, and leakage mitigation. As reservoirs become increasingly complex and the demand for sustainable energy grows, continued innovation in gel technologies is crucial. This editorial introduces a Special Issue titled “Advances and Applications of Polymer Gels for Subsurface Energy and Storage,” which compiles seven original research articles exploring recent developments in gel synthesis, characterization, and applications. The featured studies highlight the versatility of polymer gels, including nanoparticle-reinforced composites, foam–gel hybrids, recrosslinkable preformed particle gels, and advanced fracturing fluids. The contributions address key challenges across CO_2_-enhanced oil recovery, heavy oil production, low-permeability reservoir fracturing, and combined enhanced oil recovery strategies. The findings demonstrate ongoing efforts to tailor gel systems for harsh reservoir conditions, improve sweep efficiency, and reduce formation damage, fostering more efficient and sustainable subsurface engineering practices. This Special Issue serves as a valuable resource for researchers and practitioners advancing polymer gel technologies for energy and storage applications.

The exploration and efficient extraction of subsurface energy resources, alongside the growing need for geological storage, are the key challenges in the global transition towards more sustainable energy systems. Polymer gels have become important functional materials because of their wide range of applications, including conformance control, fluid diversion, fracturing stimulation, and leakage mitigation, among others [1,2]. Their unique ability to modify permeability, withstand harsh reservoir conditions, and transport proppants or active agents has led to their widespread application in subsurface energy and storage operations, including oil and gas production (e.g., improved oil recovery (IOR), conformance control, and drilling fluids), geothermal energy development, and CO_2_ geological storage [3,4]. Reservoir conditions are becoming increasing complex due to long-term water flooding and the growing demand for low-carbon and efficient energy production. Continued advancements in polymer gel systems are expected to improve the efficiency and environmental sustainability of subsurface operations [5].

Figure 1 briefly summarizes the applications of gels in oil and gas development [6,7]. Owing to their tunable rheological properties, controllable gelation behavior, and adaptability to diverse reservoir conditions, polymer gels have been developed for a wide range of applications, including well construction, reservoir stimulation, production optimization, conformance control, enhanced oil recovery, and, more recently, carbon and energy storage. Different applications impose distinct functional requirements, driving the development of specialized gel systems with tailored compositions and structures to meet specific engineering objectives.

This Special Issue, entitled “Advances and Applications of Polymer Gels for Subsurface Energy and Storage”, includes seven research articles covering recent developments in gel synthesis, rheological characterization, structure–property relationships, and engineering applications. Overall, these studies demonstrate the versatility of polymer gel systems—including viscoelastic polymer solutions, crosslinked gels, foam–gel hybrids, and nanoparticle-reinforced composites—and illustrate their broad applicability under different reservoir conditions and operational requirements.

In the fields of CO_2_-enhanced oil recovery (CO_2_-EOR) and heavy oil recovery, two studies investigated polymer gel systems to improve sweep efficiency under challenging reservoir conditions. In “Experimental Evaluation of a Recrosslinkable CO_2_-Resistant Micro-Sized Preformed Particle Gel for CO_2_ Sweep Efficiency Improvement in Reservoirs with Super-K Channels”, Alotibi et al. (contribution 1) evaluated a recrosslinkable CO_2_-resistant branched preformed particle gel (CO_2_-BRPPG) for conformance control during CO_2_ flooding. Using sandpack experiments, the authors showed that the gel exhibited an equilibrium swelling ratio of 76, stable plugging performance under acidic and high-salinity conditions, and a CO_2_ breakthrough pressure of 177 psi. The recrosslinking capability enabled the gel particles to maintain sealing performance after deformation, indicating their potential for controlling high-permeability/fracture channels and improving sweep efficiency during CO_2_ injection. In “Construction and Mechanism of Janus Nano-Graphite Reinforced Foam Gel System for Plugging Steam in Heavy Oil Reservoirs”, Xu et al. (contribution 2) developed a Janus nano-graphite-reinforced foam gel system for steam plugging in heavy oil reservoirs. At 250 °C and 5 MPa, the foam half-life reached 47.8 min, approximately 3.4 times that of a conventional foam system. The addition of nano-graphite improved foam stability and shear viscosity, enabling effective steam-channeling control in reservoirs with permeability differentials of up to 9.

In the field of hydraulic fracturing, two studies investigated novel polymer gel-based fracturing fluids for low-permeability reservoirs. In “Preparation and Performance Evaluation of CO_2_ Foam Gel Fracturing Fluid”, Gao et al. (contribution 3) developed a CO_2_ foam gel fracturing fluid using optimized thickening and foaming agents, which exhibited good static sand-carrying capacity and maintained a viscosity above 20 m·Pa·s after 90 min of shearing at 110 °C. The authors suggested that the system may be suitable for low-permeability, water-sensitive reservoirs while also providing opportunities for CO_2_ utilization and storage. In the second paper, “A Novel Fracturing Fluid Based on Functionally Modified Nano-Silica-Enhanced Hydroxypropyl Guar Gel”, Huang et al. (contribution 4) developed a novel nano-silica-enhanced gel system with improved thermal and shear resistance and reduced formation damage due to its pseudoplastic strong-gel behavior. Compared with conventional HPG gel, the FNG system retained a 9.5-percentage-point higher fracture conductivity, indicating its potential for stimulating low-permeability reservoirs. In addition, in “Evidence for Early-Time Spurt-Loss Dominance in Borate-Crosslinked HPG Gel Leakoff for High-Permeability Sandstone”, Li et al. (contribution 5) investigated the leakoff behavior of borate-crosslinked hydroxypropyl guar (HPG) gels in high-permeability formations for fracturing fluid evaluation using sandstone rock rather than a traditional filter-paper test. Their results showed that, in sandstone cores with permeabilities greater than 1.55–2.42 μm^2^, early-time spurt loss accounted for a substantial portion of the total fluid invasion, whereas late-time wall-building contributed less. The authors developed an empirical spurt-loss model that explicitly incorporates permeability and pressure differentials. Based on this model, zoning maps were constructed for engineering evaluation. The study provides further insight into fluid-loss behavior in ultra-high-permeability formations and may help improve the design of fluid-loss-control strategies.

In the field of enhanced oil recovery, Raupov et al. (contribution 6) investigated the viscoelastic properties of hydrolyzed polyacrylamide (HPAM) solutions and their influence on displacement efficiency in heterogeneous reservoirs in Russia. Using steady-shear and oscillatory rheological measurements, the authors evaluated the effects of molecular weight, pore size, salinity, and temperature on polymer viscoelasticity. Core flooding experiments showed that a combined injection strategy using polymers with different molecular weights increased oil recovery to 74–76% of the original oil in place (OOIP). Although the study focused on polymer solutions rather than crosslinked gels, its findings provide useful information on the rheological behavior of polymer-based materials that are widely used in gel formulation and subsurface flow control.

In the field of combination technologies for enhanced oil recovery, Zhang et al. (contribution 7) investigated an integrated method combining polymer, hydrophobic associating polymer (HAP), polymer microgel (MG), and preformed particle gel (PPG). Their results showed that the combination method could increase oil recovery by 17.79% in heterogeneous reservoirs compared with conventional methods. The study contributes to the understanding of conformance control strategies for multilayer reservoirs.

Overall, these seven contributions provide new insights into the behavior and applications of polymer gel systems in subsurface energy and storage. The studies cover a range of topics, including enhanced oil recovery, hydraulic fracturing, CO_2_ EOR and storage, and combined technology for EOR, illustrating the broad applicability of polymer gels under different conditions. They also demonstrate the ongoing efforts to improve gel performance through the incorporation of nanomaterials, hybrid foam–gel systems, and tailored polymer formulations. Together, these studies contribute to the continued development of polymer gel technologies for subsurface engineering.

We sincerely thank all the authors for their valuable contributions and the reviewers for their constructive comments, which helped improve the quality of this Special Issue. We also appreciate the editorial team of *Gels* for their professional support. We hope this Special Issue will provide a useful source for researchers and practitioners interested in polymer gels and their applications in subsurface energy and storage.

## Figures and Tables

**Figure 1:**
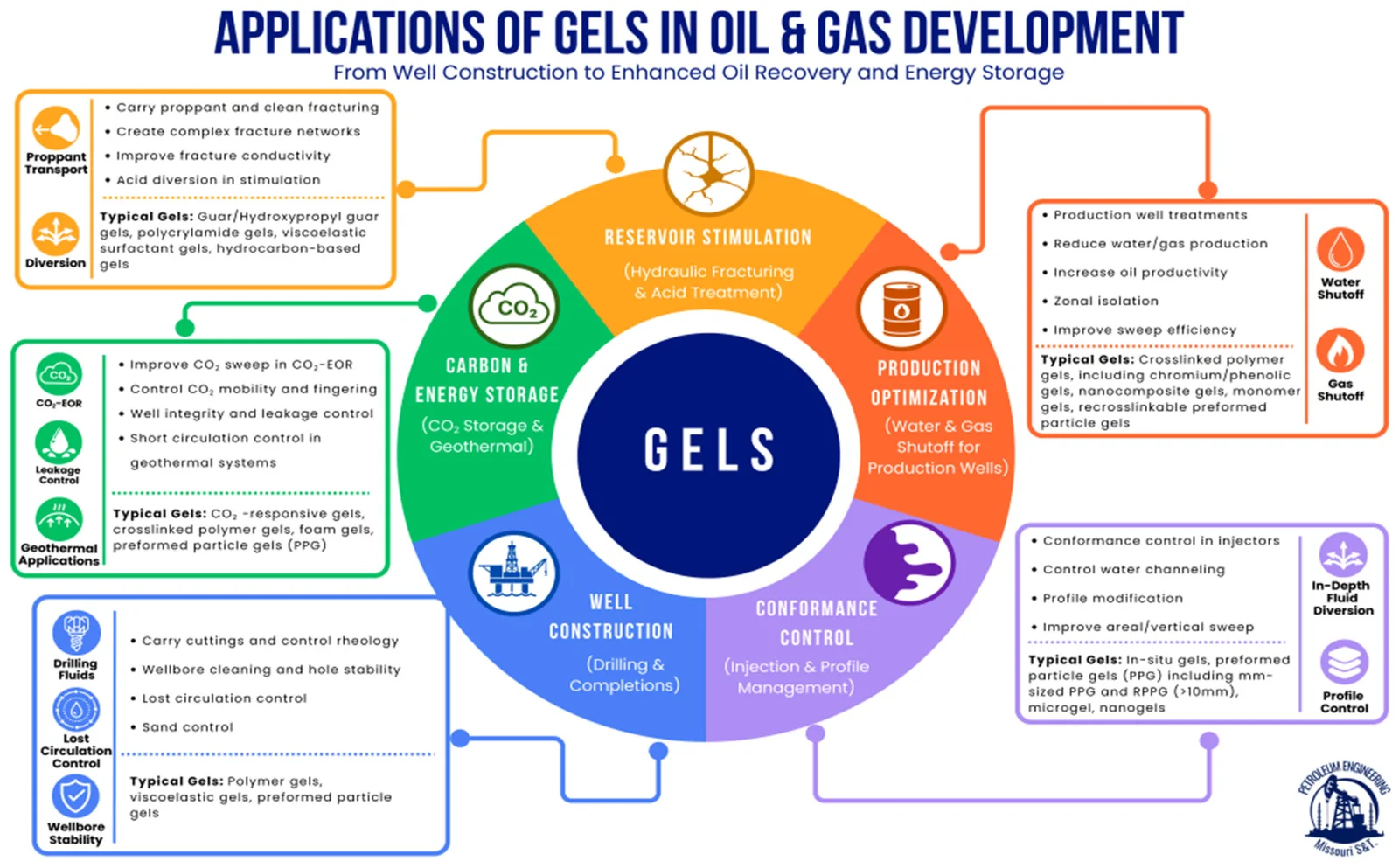
Applications of gels in oil and gas development.

## References

[1] Borhani A., Ghazi F., Akbari A., Ranjbar A., Kazemzadeh Y. (2025). A comprehensive review of advanced polymer gel technologies in enhanced oil recovery and water production control. Ore Energy Resour. Geol..

[2] El-Karsani K., Al-Muntasheri G., Sultan A., Hussein I. (2014). Gelation kinetics of PAM/PEI system. J. Therm. Anal. Calorim..

[3] Bai B., Zhou J., Yin M. (2015). A comprehensive review of polyacrylamide polymer gels for conformance control. Pet. Explor. Dev..

[4] Bashir A., Ali M., Patil S., Aljawad M., Mahmoud M., Al-Shehri D., Hoteit H., Kamal M. (2024). Comprehensive review of CO_2_ geological storage: Exploring principles, mechanisms, and prospects. Earth-Sci. Rev..

[5] Yu J., Li X., Qiao R., Zheng L., Khan N., He H., Peng Z., Yu Y. (2025). CO_2_-responsive gels for enhanced oil recovery and carbon sequestration: A comprehensive review of design and applications. J. Environ. Chem. Eng..

[6] Telin A., Lenchenkova L., Yakubov R., Poteshkina K. (2023). Application of Hydrogels and Hydrocarbon-Based Gels in Oil Production Processes and Well Drilling. Gels.

[7] Pu J., Bai B., Alhuraishawy A., Schuman T., Chen Y., Sun X. (2019). A recrosslinkable preformed particle gel for conformance control in heterogeneous reservoirs containing linear-flow features. SPE J..

